# Personality, purpose, and wellbeing: a comparison of Iranian and U.S. telecommuters during the COVID-19 pandemic

**DOI:** 10.3389/fpsyg.2025.1651968

**Published:** 2026-01-22

**Authors:** Elnaz Abaei, Zahra Ameli Renani, David Lester

**Affiliations:** 1 Department of Human Development and Family Studies, Iowa State University, Ames, IA, United States; 2 Department of Counselor Education and Counseling Psychology, Western Michigan University, Kalamazoo, MI, United States; 3 Psychology Program, Stockton University, Galloway, NJ, United States

**Keywords:** COVID-19 pandemic, MBTI, personality, sense of purpose, telecommuting (telework/remote), work wellbeing

## Abstract

The present study compared employees in Iran and the United States to examine whether personality traits predicted sense of purpose and wellbeing during telecommuting in the COVID-19 pandemic. A total of *N* = 142 participants were included (73 Iranian employees in Iran; 69 U.S. employees in the United States). Personality was assessed using the Myers–Briggs Type Indicator (MBTI), and purpose and wellbeing were measured with validated scales. Descriptive analyses showed that Iranian participants scored higher on the Sensing dimension, with no other mean differences or gender effects. Regression analyses indicated that Extraversion significantly predicted sense of purpose (β = 0.387, *p* < 0.01, *R*^2^ = 0.305) and wellbeing (β = 0.515, *p* < 0.001, *R*^2^ = 0.260) among Iranian workers, whereas Judging marginally predicted purpose (β = 0.263, *p* < 0.10, *R*^2^ = 0.128) and wellbeing (β =0.227, *p* < 0.10, *R*^2^ = 0.136) among U.S. workers. Overall, personality–outcome associations were modest in both groups, and mean levels of purpose and wellbeing did not differ across countries. This study contributes to cross-cultural telework research by highlighting culturally specific links between personality and psychological functioning during a global crisis.

## Introduction

A sense of purpose has been described as a cognitive process that defines life goals and provides personal meaning (McKnight and Kashdan, 2009). Damon et al. (2003) defined purpose as a stable and generalized intention to accomplish something that is meaningful to the self and of consequence to the world beyond the self. This conceptualization highlights three essential components of purpose: (1) being future-oriented, (2) engaging in meaningful activities with intention, and (3) striving to make a contribution beyond oneself (Malin, 2015).

Purpose can be understood through both developmental and psychological lenses. From a lifespan developmental perspective, purpose emerges as individuals grow and develop an awareness of the long-term consequences of their actions. Psychologically, it is considered a key facet of wellbeing and a marker of adaptive development (Burrow and Hill, 2020).

Wellbeing is a multifaceted construct encompassing psychological, social, and emotional dimensions, and it is closely linked to a sense of purpose and adaptive functioning. Research indicates that individuals with a strong sense of purpose experience higher life satisfaction, greater resilience under stress, and better social and relational outcomes (McKnight and Kashdan, 2009; Pfund et al., 2024; Wang et al., 2021). Longitudinal studies suggest that purpose can actively shape personality development over time, enhancing traits such as conscientiousness, extraversion, and agreeableness while reducing neuroticism (Joshanloo, 2024). During the COVID-19 pandemic, wellbeing was particularly influenced by work conditions, social support, and coping resources: flexible or hybrid work arrangements, psychological capital, self-compassion, and connectedness were all associated with higher wellbeing, whereas prolonged stress and disrupted routines threatened it (Wang et al., 2021; Riaz et al., 2024; Samios et al., 2021; VanRoo et al., 2023). Cultural factors further shape wellbeing, with collectivist contexts emphasizing relational quality and social harmony, which can buffer stress and support a meaningful sense of purpose (Suar et al., 2019, 2021). These findings underscore that wellbeing arises from an interaction of individual traits, social resources, and situational contexts, highlighting the importance of considering both personal and cultural factors in research on adaptive functioning during crises.

McKnight and Kashdan (2009) conceptualized purpose as a central, self-organizing life aim that directs goals, regulates behavior, and provides a sense of meaning. They likened it to a compass, guiding individuals in allocating their resources and making daily decisions aligned with broader life goals. While goals may offer direction, purpose provides the underlying structure and coherence that gives those goals lasting significance (Emmons, 1999; McKnight and Kashdan, 2009).

Although much research on personality and wellbeing relies on the Big Five framework, the Myers–Briggs Type Indicator (MBTI) offers a complementary perspective that emphasizes how individuals perceive information and make decisions—processes highly relevant during stress and uncertainty (Ma, 2025). MBTI dimensions capture cognitive and behavioral tendencies that may influence purpose and wellbeing in remote work contexts. For instance, Intuitive types may sustain purpose by linking current challenges to long-term goals, while Sensing types may focus on concrete, immediate tasks. Feeling types often maintain wellbeing through emotional connection, whereas Thinking types may derive meaning from problem-solving. Judging types, who value structure and predictability, may preserve purpose under disruption, while Perceiving types may find purpose through adaptability.

From an evolutionary standpoint, a sense of purpose has been associated with more efficient resource use and longer lifespan (Cichon, 1997). Individuals who possess a strong sense of purpose are believed to respond more adaptively to environmental stressors, thereby enhancing their chances of survival and wellbeing.

Recent longitudinal research also suggests that purpose is not merely a byproduct of personality traits but may actively shape personality development over time. Using a nationally representative American sample of over 11,000 adults, Joshanloo (2024) found that increases in sense of purpose predicted later increases in openness, conscientiousness, extraversion, and agreeableness, and decreases in neuroticism. Interestingly, the reverse was not true. Changes in personality traits did not predict future changes in purpose. These findings suggest that intentionally cultivating a strong sense of purpose may initiate positive personality change through enhanced self-regulation, goal-directed behavior, and improved wellbeing.

Purpose has also been found to play a meaningful role in positive development during emerging adulthood. Hill et al. (2015) developed a brief measure of purpose in life and demonstrated that, even after controlling for Big Five personality traits, purpose predicted higher levels of wellbeing, better self-image, and lower levels of delinquency in American and Canadian emerging adults. More recently, Beatty et al. (2025) found that university students with a stronger sense of purpose reported greater self-management skills (part of the broader set of social-emotional-behavioral skills) as well as higher life satisfaction, college satisfaction, and student connectedness. These findings support the idea that purpose is intricately linked to the psychosocial resources that promote success and satisfaction in higher education contexts.

Research in occupational settings supports this dynamic view of purpose. In a study comparing scientists and non-scientists, Sato (2016) found that scientists exhibited higher levels of openness, self-direction, subjective happiness, and sense of purpose than non-scientists. These findings suggest that individuals whose traits and values align with their professional activities may be more likely to experience wellbeing and life meaning. Specifically, traits such as curiosity and creativity, along with values emphasizing autonomy and intellectual exploration, appear to reinforce both purpose and happiness in highly cognitively demanding roles. Research indicates that both individual factors, such as competitiveness (Abaie et al., 2021), and organizational conditions, such as training and telecommuting experience (Khodaparasti and Garbollagh, 2023), influence employees' sense of purpose, wellbeing, and productivity in remote work contexts.

Beyond individual functioning, purpose is also positively associated with interpersonal wellbeing. In two large studies of adults in romantic relationships, Pfund et al. (2024) found that a greater sense of purpose was associated with higher relationship satisfaction, commitment, and investment. More recent longitudinal research by Pfund and Hill (2022) extended this work by showing that sense of purpose predicted the likelihood of maintaining a romantic relationship over a 3-month period. These findings suggest a reciprocal dynamic in which romantic relationships may help cultivate purpose, and individuals with higher purpose may be more likely to remain in relationships.

Emerging research also underscores the role of adversity in shaping one's sense of purpose, particularly through the lens of discrimination and intergenerational effects. Wolk et al. (2024) examined how experiences of discrimination were related to sense of purpose across two generations in the St. Louis Personality and Aging Network study. Results revealed mixed evidence for a direct link between discrimination and sense of purpose, with weaker effects observed for Black adults compared to White adults. Importantly, while no intergenerational transmission of purpose was observed, experiences of discrimination did show intergenerational continuity, suggesting a potential cycle of marginalization that may indirectly influence the development of purpose.

Purpose has also been closely linked with social support and loneliness. In a large, nationally representative Swiss sample of over 2,300 adults, Hill et al. (2023) found that purpose was positively associated with both received and provided social support and negatively associated with loneliness. While the association between purpose and loneliness remained stable across the adult lifespan, the strength of the association between purpose and support decreased with age.

Meaning in life has long been central to existential psychology and psychiatry. (Frankl 1959), (1967), (1984) proposed that humans are fundamentally driven by a “will to meaning,” or the innate desire to find value and purpose even under the most challenging conditions. He emphasized that meaning can be discovered in all circumstances, including suffering, and that this pursuit is essential to psychological health.

However, the maintenance of meaning is vulnerable during periods of uncertainty or crisis. According to the Meaning Maintenance Model (MMM; Heine et al., 2006), individuals strive to sustain coherent systems of meaning that encompass domains such as self-worth, certainty, social connectedness, and symbolic continuity. When disruption occurs in one domain, compensatory reinforcement in another may help restore psychological equilibrium (Van Tongeren and Green, 2010).

Disruptions in meaning can produce existential distress, often manifesting as anxiety, hopelessness, or guilt (Tillich, 1952; Frankl, 1959). van Selm and Dittmann-Kohli (1998) distinguished between motivational meaninglessness, characterized by a lack of direction and goals, and cognitive meaninglessness, which involves disconnection from the self, others, or society. In later adulthood, such disconnection may take the form of despair, particularly if individuals cannot reconcile their life narratives with a sense of value or fulfillment (Erikson, 1963; Erikson et al., 1986; Butler, 1977).

The COVID-19 pandemic significantly disrupted everyday life, particularly through the widespread transition to telecommuting. This shift in work structure and social interaction likely impacted individuals' experience of purpose and wellbeing. More recent findings by VanRoo et al. (2023) show that college students reported high stress and fluctuating levels of grit and purpose, normalizing chronic stress over time. Reich et al. (2023) found that a strong sense of purpose buffered the effects of financial strain on caregiver mental health and children's behavioral outcomes. Saraniemi et al. (2023) described how Finnish social workers navigated a “liminal space,” where blurred work-life boundaries opened opportunities to reframe professional meaning. Samios et al. (2021) found that self-compassion, especially the sense of common humanity, moderated the negative relationship between pandemic-related stress and meaning in life. Finally, Fan and Moen (2023) showed that ongoing remote or hybrid work arrangements were linked to greater wellbeing compared to full returns to the office, highlighting how flexible work contexts may help sustain a sense of purpose in the post-pandemic era. Together, these studies illustrate how purpose can act as both a protective resource and an outcome of adaptive coping in times of crisis.

Cultural and situational contexts further clarify how personality shapes wellbeing and purpose. In collectivist settings such as India, subjective wellbeing is strongly predicted by emotional stability and relationship quality, reflecting cultural values of harmony and belonging (Suar et al., 2019), and happiness often emerges through interpersonal and prosocial engagement (Suar et al., 2021). During the COVID-19 lockdown, psychological capital and internal locus of control also helped buffer distress (Alat et al., 2021). These patterns suggest that connectedness and resilience play central roles in collectivist cultures, helping explain why extraversion supported purpose and wellbeing among Iranian workers, whereas judging modestly predicted these outcomes among American workers. Prior cross-national work comparing Iranian and U.S. employees has similarly shown that cultural context moderates how work-related traits relate to functioning, such as differences in the link between competitiveness and organizational commitment (Abaie et al., 2021). Despite this growing literature, few studies have directly examined personality–purpose associations across culturally distinct telecommuting samples during a global crisis. By comparing Iranian and U.S. telecommuters during the COVID-19 pandemic, the present study integrates personality psychology, cultural context, and crisis-driven remote work to address this gap.

The purpose of the present study was to examine the association between sense of purpose and personality traits in a two countries (Iran and the United States) during the COVID-19 pandemic when employees had to work from home using the Internet.

To make these objectives explicit, we formulated the following research questions and hypotheses:

Research Question 1: How are MBTI personality dimensions associated with sense of purpose and wellbeing among telecommuters in Iran and the United States?

Hypothesis 1: Extraversion will be positively associated with sense of purpose and wellbeing for Iranian telecommuters, reflecting the value of social engagement in collectivist contexts.

Hypothesis 2: Judging will be positively associated with sense of purpose and wellbeing for U.S. telecommuters, reflecting the importance of structure and planning under uncertainty.

Exploratory Question: How do the other MBTI dimensions (Sensing–Intuition, Thinking–Feeling) relate to purpose and wellbeing across cultural contexts?

## Method

### Participants

Seventy-three employees from Iran and 69 from the United States participated in the study. The Iranian sample had a higher proportion of women (75.3%) than the American sample (58.0%), χ^2^(df = 1, *N* = 142) = 4.86, *p* < 0.01. Iranian participants were also older (*M* = 37.64, *SD* = 8.02) than American participants (*M* = 33.45, *SD* = 3.08), *t*(df = 140) = 4.07, *p* < 0.001. Education level was coded as follows: 1 = *some schooling*, 2 = *high school diploma*, 3 = *bachelor's degree*, 4 = *master's degree*, and 5 = *PhD*. A significantly higher proportion of American participants held PhD degrees (71.0%) compared to Iranian participants (4.1%), χ^2^(df = 4, *N* = 142) = 89.72, *p* < 0.001. Participants in both countries were recruited online during the COVID-19 pandemic. Recruitment links to online questionnaires were distributed through WhatsApp and Telegram to individuals known to be telecommuting.

### Ethics statement

All participants provided informed consent prior to participation. They were presented with a description of the study procedures and asked to indicate their consent by clicking “I agree and proceed to the survey” before completing the questionnaire. The survey included questions related to personality, sense of purpose, and wellbeing and required approximately 15–20 min to complete. All data were collected anonymously and de-identified to protect participant confidentiality.

Data collection in Iran occurred in the absence of a local IRB. Procedures were conducted in accordance with its ethical standards and internationally accepted guidelines for human subjects research. These procedures ensured that participation was voluntary, confidential, and ethically compliant despite the lack of a local IRB. Data collection took place during the COVID-19 pandemic in 2021.

### Measures

#### Sense of purpose questionnaire

The Sense of Purpose Questionnaire (Crumbaugh and Maholick, 1969) includes 20 items rated on a 7-point Likert scale ranging from 1 (completely bored) to 7 (exuberantly enthusiastic). Higher scores reflect a greater sense of purpose. Several items are phrased negatively (e.g., “I am usually bored,” “My life is empty”), and we reverse-scored these so that higher values consistently indicate higher purpose in life. We computed a total score by summing all items, yielding a theoretical range of 20–140. Sample items include “I am usually: bored to enthusiastic,” “My personal existence is: meaningless to purposeful and meaningful,” and “In life I have: no goals to very clear goals and aims.” Cronbach's alpha was 0.94 for the Iranian sample and 0.90 for the American sample.

#### WellBeing questionnaire

The WellBeing Questionnaire (Zheng et al., 2015) contains 18 items rated on a 5-point Likert scale ranging from 1 (strongly disagree) to 5 (strongly agree). All items are scored in the positive direction, and no reverse scoring is required. Higher scores indicate greater subjective wellbeing. We summed all items to create a total wellbeing score with a theoretical range of 18–90. Sample items include “I feel satisfied with my life,” “Most of the time, I do feel real happiness,” and “I feel I have grown as a person.” Cronbach's alpha was 0.89 for the Iranian sample and 0.86 for the American sample.

#### Myers–Briggs type indicator (MBTI)

The MBTI (Myers, 1962) consists of 86 dichotomous (two-choice) items. A sample item is: “At a party, do you: (a) interact with many, including strangers, or (b) interact with a few, known to you?” The version used was a scaled version developed in Iran (Elyasi et al., 2023). For the Iranian sample, reliability coefficients computed on continuous scores ranged from 0.71 to 0.82 across the four dimensions. For the U.S. sample, reliability coefficients ranged from 0.78 to 0.83.

Because the MBTI is a typological instrument designed to classify individuals into dichotomous preference categories rather than to represent continuous latent constructs, traditional measurement invariance testing frameworks [e.g., multi-group confirmatory factor analysis (CFA)] are not directly applicable. Accordingly, formal tests of measurement invariance across the two national samples were not conducted. To address this limitation, cross-national comparisons involving MBTI dimensions are interpreted as descriptive and exploratory rather than as evidence of strict cross-cultural equivalence.

## Results

To address the research questions and test the hypotheses, separate multiple regression analyses were conducted for Iranian and U.S. telecommuters predicting Sense of Purpose and WellBeing from the four MBTI personality dimensions (Tables 1–3).

**Table 1:** Mean scores on psychological measures by country.

**Questionnaire**	**Iran *M (SD)***	**USA *M (SD)***	***t*-value**
Sense of purpose	111.02 (20.31)	108.80 (15.01)	0.70
Wellbeing	68.16 (10.89)	68.49 (9.43)	0.19
**Myers–Briggs**			
E (Extraversion)	58.14 (24.28)	56.30 (23.62)	0.46
S (Sensing)	55.77 (16.61)	47.07 (18.07)	2.99^*^
T (Thinking)	67.22 (16.85)	65.52 (17.93)	0.58
J (Judging)	78.53 (15.01)	77.41 (21.39)	0.32

^*^p < 0.10.

**Table 2:** Regression coefficients predicting sense of purpose (standardized betas).

**Predictor**	**Iran β**	**USA β**
Age	+0.092	+0.154
Sex	+0.108	+0.164
Education	−0.068	+0.051
E	+0.387^**^	+0.074
S	−0.024	−0.088
T	+0.111	+0.024
J	+0.160	+0.263^*^
*R* ^2^	0.305	0.128

^*^p < 0.10.

^**^p < 0.01.

**Table 3:** Regression coefficients predicting wellbeing (standardized betas).

**Predictor**	**Iran β**	**USA β**
Age	+0.048	+0.124
Sex	−0.045	+0.126
Education	−0.148	+0.036
E	+0.515^***^	+0.168
S	+0.016	−0.137
T	+0.045	+0.010
J	+0.034	+0.227^*^
*R* ^2^	0.260	0.136

^*^p < 0.10.

^***^p < 0.001.

Hypothesis 1: Extraversion predicts greater purpose and wellbeing among Iranian telecommuters.

In the Iranian sample, Extraversion emerged as the strongest and only consistent personality predictor of both Sense of Purpose (β =0.387, *p* < 0.01) and WellBeing (β =0.515, *p* < 0.001), supporting Hypothesis 1. Other MBTI dimensions (Sensing–Intuition, Thinking–Feeling, and Judging) did not significantly predict outcomes. The overall models accounted for 30.5% of variance in Sense of Purpose and 26.0% in WellBeing (*R*^2^ = 0.305 and 0.260, respectively), indicating substantial trait relevance in this collectivist context.

Hypothesis 2: Judging predicts greater purpose and wellbeing among U.S. telecommuters.

For U.S. telecommuters, Judging modestly predicted Sense of Purpose (β =0.263, *p* < 0.10) and WellBeing (β =0.227, *p* < 0.10), supporting Hypothesis 2. No other MBTI dimensions were significant predictors. The models explained 12.8% of variance in Sense of Purpose and 13.6% in WellBeing (*R*^2^ = 0.128 and 0.136), suggesting a smaller, though meaningful, influence of personality traits in this individualistic sample.

A confirmatory factor analysis (CFA) was conducted separately for Iranian and U.S. telecommuters to evaluate the factor structure of the Sense of Purpose (PIL) scale. In both samples, a single-factor model demonstrated acceptable overall fit (Iran: χ^2^(170) = 286.45, *p* < 0.001, CFI =0.93, TLI =0.92, RMSEA =0.06, 90% CI [0.05, 0.07], SRMR = 0.06; U.S.: χ^2^(170) = 271.18, *p* < 0.001, CFI = 0.94, TLI = 0.93, RMSEA = 0.05, 90% CI [0.04, 0.06], SRMR = 0.05). Standardized factor loadings ranged from 0.53 to 1.77 in both samples and were statistically significant (all ps < 0.001).

### Exploratory analyses: relations of sensing–intuition and thinking–feeling to purpose and wellbeing

Neither Sensing–Intuition nor Thinking–Feeling significantly predicted outcomes in either country. Although Iranian participants scored higher on Sensing descriptively (*M* = 55.77 vs. 47.07, *t* = 2.99, *p* < 0.01), this trait did not significantly relate to purpose or wellbeing. These null findings suggest that these dimensions may be less relevant to telecommuters' psychological outcomes or that their effects depend on situational factors not captured in this study. Prior to interpreting regression results, we examined model assumptions to ensure validity. Multicollinearity was assessed using Variance Inflation Factors, all of which were below 2.5, indicating no problematic overlap among predictors. Residuals were inspected for normality and homoscedasticity using Q–Q plots and scatterplots of standardized residuals, revealing no major deviations. Influential cases were identified via Cook's distance and leverage statistics, with no cases exceeding recommended thresholds.

### *Post-hoc* power analysis

A *post-hoc* power analysis was conducted using α = 0.05 and observed effect sizes (*f*^2^) for each regression model, following Cohen's benchmarks (small = 0.02, medium = 0.15, large = 0.35). For Iranian telecommuters, Extraversion predicting Sense of Purpose yielded *f*^2^ = 0.44 and Extraversion predicting WellBeing yielded *f*^2^ = 0.35, both reflecting large effects. For U.S. telecommuters, Judging predicting Sense of Purpose yielded *f*^2^ = 0.15 and Judging predicting WellBeing yielded *f*^2^ = 0.12, reflecting small-to-medium effects. Corresponding statistical power was 0.811 for small effects, 0.984 for medium effects, and >0.99 for large effects, indicating that the study was sufficiently powered to detect meaningful associations, though very small effects may have gone undetected.

To assess the robustness of our regression results, we conducted bootstrapping with 5,000 resamples separately for the Iranian (*n* = 73) and U.S. (*n* = 69) samples. Bootstrapped confidence intervals for all significant predictors (Extraversion in Iran; Judging in the U.S.) were consistent with the original regression results, and no previously non-significant predictors became significant. These analyses suggest that the main findings are stable and not driven by sample composition or influential cases.

## Discussion

This study examined whether MBTI personality traits predicted sense of purpose and wellbeing among Iranian and American employees working remotely during the COVID-19 pandemic. Prior literature highlights the central role of purpose in guiding behavior, regulating emotions, and supporting long-term wellbeing (McKnight and Kashdan, 2009; Burrow and Hill, 2020). Our findings contribute to this literature by showing that personality–purpose associations differed across cultural contexts and were largely limited in scope. Consistent with Hypothesis 1, Extraversion significantly predicted both sense of purpose and wellbeing among Iranian workers. Likewise, consistent with Hypothesis 2, Judging showed modest but marginally significant associations with these outcomes among American workers. The remaining traits—Sensing–Intuition and Thinking–Feeling—did not predict either outcome in either group, aligning with our exploratory expectations.

The current findings highlight that the predictive value of MBTI personality dimensions on purpose and wellbeing is context-dependent. Extraversion strongly predicted both outcomes among Iranian telecommuters, consistent with collectivist cultural norms that emphasize social engagement and relational connectedness, whereas Judging modestly predicted outcomes among U.S. telecommuters, reflecting individualistic tendencies favoring structure and goal-directed behavior. Notably, although Iranian participants scored higher on Sensing, this trait did not significantly predict purpose or wellbeing, suggesting that trait expression may be overshadowed by situational demands during disruptive periods such as the COVID-19 pandemic. The *R*^2^ values indicate that personality accounted for a substantial portion of variance in Iranian outcomes (26%−31%) but less so for American outcomes (13%−14%), reinforcing the idea that cultural and situational contexts modulate the influence of personality on adaptive functioning. These results underscore the importance of integrating cultural frameworks and situational considerations when interpreting personality–wellbeing associations, rather than relying solely on trait-based predictions.

Cultural and situational contexts further illuminate these patterns. Research among Indian millennials shows that subjective wellbeing functions as a hierarchical construct and is most strongly predicted by emotional stability and the quality of personal relationships, highlighting the collectivist emphasis on harmony and belonging (Suar et al., 2019). Subsequent work found that millennials' happiness predominantly resides in interpersonal relationships and prosocial or expressive activities, again highlighting culturally rooted pathways to purpose-driven wellbeing (Suar et al., 2021). During the COVID-19 lockdown, psychological capital—a composite of hope, efficacy, resilience, and optimism—and internal locus of control were shown to mitigate psychological distress through positive affect balance, illustrating the protective role of inner resources and adaptive coping in times of global crisis (Alat et al., 2021). Taken together, these findings suggest that in collectivist settings, wellbeing and purpose are maintained through relational connectedness, emotional stability, and resilient self-regulation—patterns that help explain why extraversion enhanced Iranian workers' sense of purpose and wellbeing, whereas judging modestly benefited American workers by aligning with individualistic, goal-driven coping styles.

While extraversion appeared especially beneficial in the Iranian context, American participants high in the judging trait—which reflects a preference for structure, organization, and decisiveness—reported marginally greater sense of purpose and wellbeing. This is consistent with the Meaning Maintenance Model (Heine et al., 2006), which posits that individuals respond to disruptions in coherence (such as those caused by the pandemic and remote work) by reinforcing other domains of meaning. Structured individuals may have coped more effectively by establishing clear work boundaries and routines, thereby mitigating existential distress (Van Tongeren and Green, 2010) and sustaining psychological equilibrium.

The limited predictive value of other personality traits stands in contrast to longitudinal evidence showing that purpose itself may influence personality development over time, rather than being a mere byproduct of stable traits (Joshanloo, 2024). This supports a dynamic perspective in which purpose is shaped by situational, cultural, and developmental factors, especially during times of disruption.

Interestingly, although sensing was significantly higher in Iranian participants, it did not predict either sense of purpose or wellbeing. This may reflect what (Frankl 1959), (1984) described as the vulnerability of meaning during uncertain conditions—where even trait-like tendencies may become less relevant. In both cultural contexts, the pandemic represented a rupture in daily life that likely overshadowed the stable influence of most personality dimensions.

The similar levels of purpose and wellbeing across countries are also notable, particularly given differences in education, age, and gender. This convergence may reflect the pandemic's universal impact on work and meaning systems, as proposed by Saraniemi et al. (2023) and Fan and Moen (2023). Despite differing cultural contexts, participants may have encountered comparable challenges in maintaining purpose while working remotely. These findings support previous work highlighting that purpose is not only tied to individual traits but also deeply embedded in broader social and occupational contexts (Sato, 2016; Pfund et al., 2024).

Moreover, while previous research suggests that purpose is positively associated with interpersonal resources (Hill et al., 2023) and buffers the effects of adversity (Reich et al., 2023), our results suggest that in acute and highly disruptive conditions, the influence of individual differences may be attenuated. This also raises the possibility that coping processes such as self-compassion (Samios et al., 2021) or meaning reconstruction (VanRoo et al., 2023) may serve as stronger predictors of wellbeing than trait-based dispositions during crises.

### Limitations and future directions

This study has several limitations. First, the sample size was modest, which reduces statistical power and limits generalizability. Second, there were notable demographic imbalances: 71% of U.S. participants held doctoral degrees, compared with 4.1% in the Iranian sample, while the Iranian sample included more women and slightly older participants. These differences could have confounded the observed associations between MBTI traits, sense of purpose, and wellbeing.

We considered conducting sensitivity analyses to examine the potential influence of sample composition—such as excluding PhD participants or matching subsamples by education, age, or gender. However, due to the sample size, such analyses were not feasible. Excluding PhD participants alone would have removed 51 of 152 cases, substantially reducing statistical power and compromising regression stability. We therefore acknowledge that sample composition and potential measurement limitations of the MBTI may have influenced the results.

Moreover, the MBTI, though widely recognized and practical for applied settings, is not as psychometrically robust as the Big Five model, which may explain some null findings. We used a Persian version translated and adapted via standard forward–backward procedures (Elyasi et al., 2023), which has acceptable psychometric properties in Iranian samples. However, published cross-cultural measurement invariance analyses comparing the Persian and U.S. versions remain limited, so mean-level comparisons should be interpreted cautiously.

Additionally, future research should address limitations related to translation and cultural validity. While we used forward–backward translation for the Persian MBTI and other measures, potential subtle cultural biases may have influenced responses. Longitudinal and mixed-method designs are recommended to capture changes in purpose and wellbeing over time, and to explore mediators and moderators such as social support, coping strategies, and work-related stress. Testing measurement invariance across cultures would further strengthen confidence in cross-national comparisons and clarify whether constructs operate equivalently in diverse contexts.

Another potential limitation is non-response and recruitment bias. Recruitment through social networks and messaging apps may have oversampled highly connected or more educated telecommuters, limiting representativeness. Future research could mitigate this by partnering with employers to access broader employee populations or using random sampling within industries to achieve more balanced and generalizable samples.

Future research should aim to address these limitations by recruiting larger, demographically balanced cross-cultural samples. Longitudinal panel designs with measurement invariance testing are recommended to clarify cross-cultural comparability. Multi-site sampling with stratified recruitment by education, age, and gender would help balance sample composition and improve generalizability. Integrating both MBTI and Big Five frameworks, along with potential mediators such as coping styles, job demands, and social support, could provide a more comprehensive understanding of the mechanisms linking personality to purpose and wellbeing.

### Practical implications

The present findings also suggest actionable strategies for organizations managing remote employees. For telecommuters in collectivist contexts or teams with highly extraverted employees (e.g., Iranian workers), organizations may consider facilitating social interaction and virtual networking opportunities, such as structured online check-ins, peer mentoring, or collaborative platforms, to help employees maintain connection, purpose, and wellbeing. For employees with a preference for Judging—particularly in individualistic contexts like the U.S.—organizations can support structured routines and clear workflows. Tools for planning, project tracking, and goal-setting may help these employees maintain purpose and reduce stress associated with ambiguity in remote work.

Finally, the limited predictive influence of other MBTI traits highlights the importance of flexible, individualized approaches. Managers should consider employees' personality preferences alongside cultural and situational factors, offering optional interventions—such as workshops on time management, resilience, or meaning-focused coping—that support wellbeing across diverse personality profiles.

## Conclusion

In summary, while much of the existing literature emphasizes strong links between personality and purpose (McKnight and Kashdan, 2009; Emmons, 1999; Hill et al., 2015), our findings suggest these relationships are highly context-dependent. In this sample, Extraversion predicted both purpose and wellbeing in Iranian telecommuters but not in the U.S., highlighting cultural differences in how social engagement supports meaning during remote work. Similarly, Judging modestly predicted purpose and wellbeing among U.S. telecommuters, suggesting that structured, goal-oriented tendencies may buffer uncertainty in individualistic work contexts. These results underscore the importance of considering cultural and situational moderators when examining personality–purpose links and suggest that interventions to enhance wellbeing should be tailored to both personality traits and cultural context, such as fostering social connectivity for extraverted employees or supporting structured routines for those high in Judging.

## Data Availability

The original contributions presented in the study are included in the article/Supplementary material, further inquiries can be directed to the corresponding author.
